# Biomechanical evaluation of the novel assembled internal fixed system in C2–C3 anterior cervical discectomy and fusion: a finite element analysis

**DOI:** 10.1186/s13018-024-04567-5

**Published:** 2024-02-01

**Authors:** Hu Chen, Hao Sun, Lu Cao, Hong Xia, Qiang Tu

**Affiliations:** 1 The First School of Clinical Medicine, Southern Medical University, Guangzhou, 510515 Guangdong China; 2 Department of Orthopedic, People’s Liberation Army General Hospital of Southern Theatre Command, Guangzhou, 510010 Guangdong China; 3 Guangzhou University of Chinese Medicine, Guangzhou, 510006 Guangdong China; 4 Department of Dermatology, People’s Liberation Army General Hospital of Southern Theatre Command, Guangzhou, Guangdong China

**Keywords:** Finite element model, Cage-plate construct, New combined cervical fusion device, Biomechanical evaluation, ACDF

## Abstract

**Background:**

To analyze and compare the biomechanical characteristics of the new combined cervical fusion device (NCCFD) and the traditional cage-plate construct (CPC) to ascertain its effectiveness in anterior cervical discectomy and fusion (ACDF) using finite element analysis.

**Methods:**

A finite element model of the cervical spine, inclusive of the occipital bone was created and validated. In the ACDF model, either CPC or NCCFD was implanted at the C2–C3 segment of the model. A pure moment of 1.0 Nm combined with a follower load of 50 N was directed onto the superior surfaces of the occipital bone to determine flexion, extension, lateral bending (left and right), and axial rotation (left and right). The range of motion (ROM), stress distribution at the bone-implant interface, and facet joint forces were investigated and compared between CPC and NCCFD systems.

**Result:**

The results showed that the ROMs of the fused levels in both models were nearly zero, and the motions of the unfused segments were similar. In addition, the maximum displacement exhibited nearly identical values for both models. The maximum stress of NCCFD screws in lateral bending and rotational conditions is significantly higher than that of the CPC, while the NCCFD model’s maximum stress remains within an acceptable range. Comparing the maximum fusion stress, it was found that the CPC experiences much lower fusion stress in anterior flexion and extension than the NCCFD, with no significant difference between the two in lateral bending and rotational states. Stress on the cage was mainly concentrated on both sides of the wings. Comparing the maximum IDP in the CPC and NCCFD, it was observed that maximum stresses rise in extension and lateral bending for both models. Lastly, stress distributions of the facet joints were generally similar across the two devices.

**Conclusion:**

NCCFD not only provides the same level of biomechanical stability as CPC but also avoids postoperative complications associated with uneven force damage to the implant. The device offers a novel surgical alternative for ACDF in C2–C3 level.

## Background

Anterior cervical discectomy and fusion (ACDF) is a widely employed surgical technique known for its high efficacy in addressing degenerative disc disease or cervical disc herniation within the cervical spine [1]. It provides direct access to remove the herniated or degenerated disc, relieving pressure on the nerves, and restoring normal functioning and symptom resolution. The traditional cage-plate construct (CPC) has gained popularity as a classical tool for ACDF surgery [2]. It provides immediate stability to the anterior column and demonstrates good biocompatibility post-surgery, leading to a relatively high bone fusion rate. While ACDF using the CPC is usually performed in the lower cervical spine levels (C3–C7), in certain cases, such as Hangman fractures, necessitate ACDF at the C2–C3 level, posing unique challenges for surgeons [3]. ACDF using CPC at the C2–C3 level has been associated with various complications. Previous review of 85 patients who underwent ACDF for upper cervical spine conditions revealed that 11% of them experienced postoperative voice changes, and 3.5% suffered from permanent vocal cord paralysis. Additionally, a prospective study involving 87 patients who underwent single or double segments ACDF showed that prevertebral soft tissue exhibited greater severity in the group undergoing surgery proximal to C5, as opposed to the group undergoing surgery distal to C5. This swelling was particularly prominent at the C2 and C3 levels and led to dysphagia [4].

During ACDF procedures utilizing the CPC, the implant's shape and the intricate anatomy at the C2–C3 level present limitations for its effective application within this segment [5]. The distinct curvature of the anterior margin of the C2 vertebra poses challenges for existing cervical bending instruments, hindering the achievement of a curvature that aligns with both the sagittal and horizontal planes of C2–C3 simultaneously [6]. Consequently, this affects the secure attachment of the titanium plate to the vertebra's anterior margin, influencing the precision and convenience of screw placement, as well as the reliability of internal fixation. Additionally, using a higher-profile titanium plate might lead to increased irritation within the soft tissues of the pharyngeal region, potentially contributing to postoperative complications.

Given these challenges, the Zero-P system presents a promising alternative for ACDF at the C2–C3 level. The Zero-P system offers several advantages, such as reducing the risk of adjacent segment disease (ASD), enhancing fusion rates, employing a minimally invasive approach, preserving range of motion, and reducing hardware-related complications [7, 8]. However, it is important to note that the Zero-P system could face limitations at C2–C3 levels due to obstruction from the mandibular bone, preventing downward implantation of the fixing nail.

Given the complexities and potential complications associated with ACDF at the C2–C3 level, further research and development of a novel internal fixed system tailored to address the anatomical challenges specific to this region would be beneficial. Such innovation could potentially enhance surgical outcomes, fusion rates, and patient comfort and recovery.

In this study, we have designed a new combined cervical fusion device (NCCFD), comprising a titanium U-shaped thin plate with three screw trajectories. This device aims to combine the strengths of both the traditional CPC model plate cage system and Zero-P implants. This study aimed to analyze and compare the biomechanical characteristics of the NCCFD and the traditional CPC to ascertain its effectiveness in ACDF using finite element (FE) analysis. Specifically, the range of motion (ROM) of the treated segment, stress distribution at the bone-implant interface, maximum intradiscal pressure (IDP) and facet joint forces were investigated for both systems.

## Methods

### Conceptual design

**Fig. 1:**
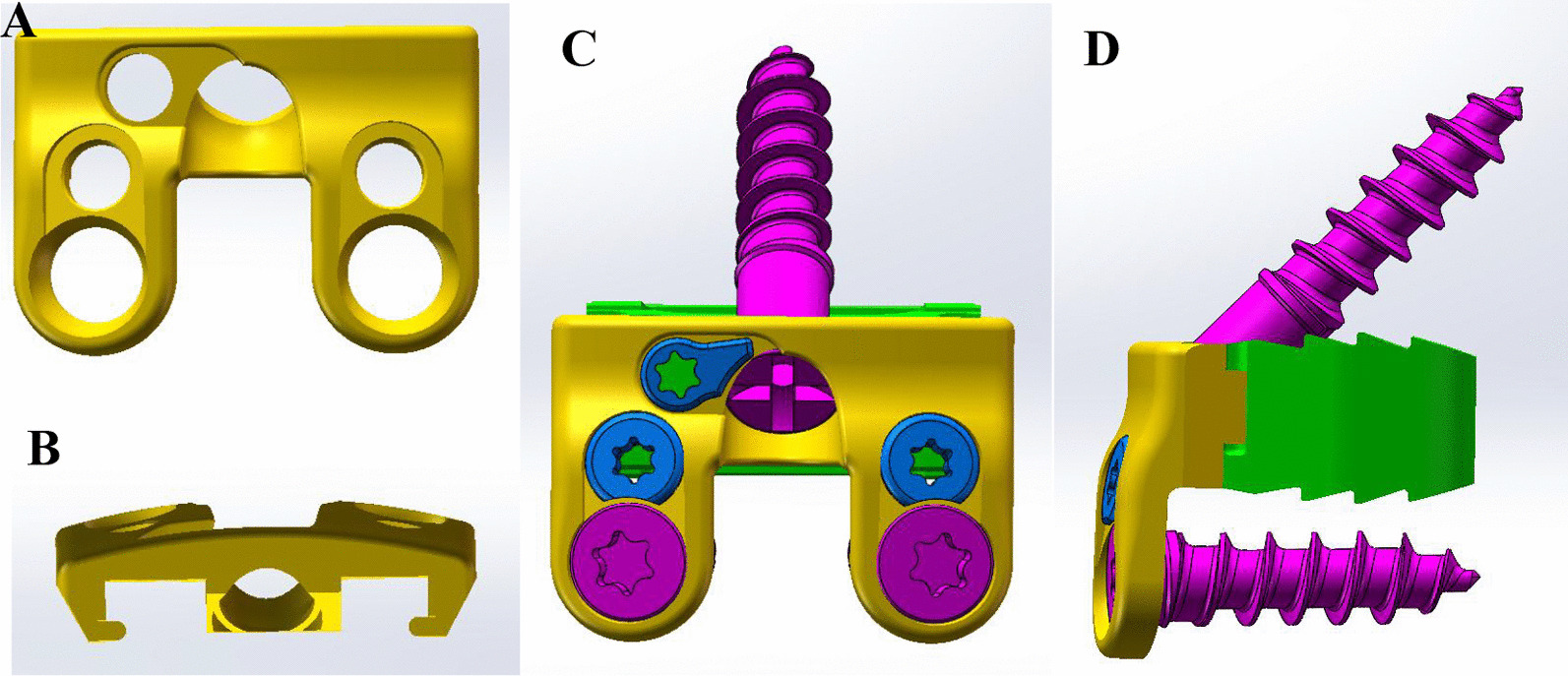
Conceptual design of new combined cervical fusion device. A front view (**A**) and top view (**B**) of the NCCFD, and a front view (**C**) and side view (**D**) of the NCCFD with screws in place

The NCCFD proposed in this study consists of two detachable components, including an anterior cervical spine plate and a polyetheretherketone (PEEK) material disc spacer. Notably, this design significantly reduces the volume of the conventional anterior cervical plate by half. The titanium plate is thoughtfully curved to better conform to the anterior edge of the C3 vertebral body. (Fig. 1A, B). For optimal stability, the plate is equipped with three screw holes, with the highest screw placed at an angle of 35°–70° to the midline of the new combined cervical fusion device. The two lower screw pathways are perpendicular to the plate to facilitate vertical nail penetration (Fig. 1C, D). Importantly, the height of the disc spacer is comparable to that of a traditional CPC interbody fusion, maintaining similar intervertebral spacing and alignment (Fig. 1D).

### FE model of the intact cervical spine

**Fig. 2:**
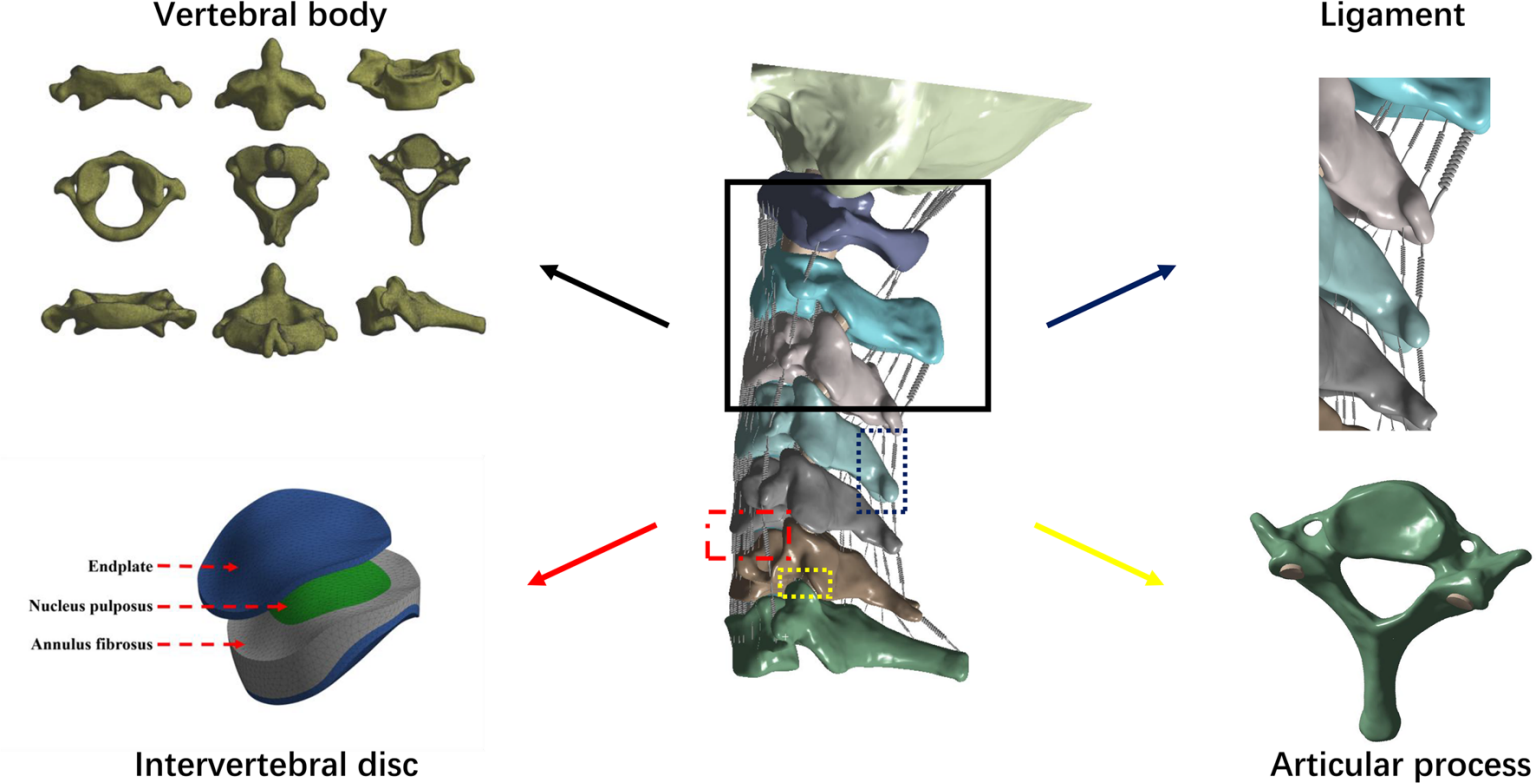
Establishment of the FE model, including vertebral body, intervertebral disc, ligament and articular process

In this study, a comprehensive FE model of the cervical spine, inclusive of the occipital bone, was meticulously developed (Fig. 2). The model was constructed based on computed tomographic (CT) images of a 37-year-old healthy male, with a height of 170 cm and a weight of 75 kg. The imaging data were loaded into Mimics software to create a bone mask and a full cervical STL triangular mesh model that included the occipital bone. The model was then imported into Geomagic software. Next, SolidWorks software was used to design features that were not clearly visible in CT scans, such as the nucleus pulposus, end plate, fibrous ring, and synovial cartilage, based on anatomical structures. Once the model was ready, it was imported into ANSYS software for meshing. To ensure the accuracy of the FE analysis, the mesh unit of this model is set to a minimum of 0.5 mm. All structures were defined using tetrahedral elements. In addition, the transverse ligament was replicated using tetrahedral elements, while all remaining ligaments of the cervical spine were integrated into the model. These ligaments were treated as nonlinear springs without affecting compression. The vertebral body and ligaments were linked through shared nodes, as were the various components of internal fixation. Nonlinear surface-to-surface contact was employed to replicate the interactions between vertebral joints.

**Fig. 3:**
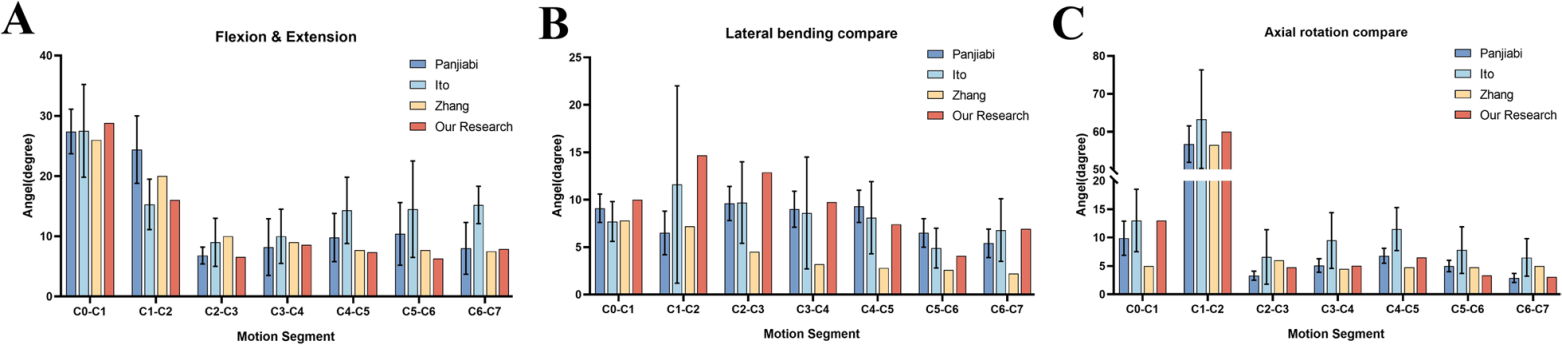
Validation of the intact cervical model, including ROM in flexion–extension (**A**), ROM in lateral bending (**B**) and ROM in axial rotation (**C**)

The complete FE model consisted of 2,218,790 elements and 3,332,459 nodes, including 7 cervical vertebrae, occiput, 5 intervertebral discs, 13 ligaments, and 7 pairs of facet joints (Table 1). To validate the accuracy of the models, the measured data were compared with the cadaveric model created by Panjabi et al. [9, 10], the finite element model created by Zhang [11] and Ito [12]. The ROMs obtained were in line with those reported in published experiments, demonstrating both value and trend consistency (Table 2, Fig. 3A–C). These findings underscore the validity of the intact FE cervical spine model.

**Table 1:** The number of elements and nodes for the cervical spine model

	Element	Node
C0	838,314	1,194,401
C1	155,190	250,308
C2	172,201	258,184
C3	126,900	192,418
C4	124,935	190,429
C5	130,864	199,496
C6	125,626	192,836
C7	160,132	241,799
C2/3	24,089	37,083
C3/4	34,443	51,962
C4/5	37,712	57,103
C5/6	43,149	64,807
C6/7	65,350	97,033
Endplates	76,908	143,494
Cartilago articularis	100,141	155,599

**Table 2:** Validation of the intact cervical model

	Panjiabi	Ito	Zhang	Our research
*Flexion and extension*				
C0–C1	27.4 ± 3.7	27.5 ± 7.7	26	28.82
C1–C2	24.4 ± 5.6	15.3 ± 4.2	20	16.02
C2–C3	6.8 ± 1.4	9.0 ± 4.0	10	6.57
C3–C4	8.2 ± 4.7	10.0 ± 4.5	9	8.59
C4–C5	9.8 ± 4.0	14.3 ± 5.5	7.7	7.34
C5–C6	10.4 ± 5.2	14.5 ± 8.0	7.7	6.3
C6–C7	8.0 ± 4.3	15.2 ± 3.1	7.5	7.86
*Lateral bending*				
C0–C1	9.1 ± 1.5	7.7 ± 2.1	7.8	10
C1–C2	6.5 ± 2.3	11.6 ± 10.4	7.2	14.69
C2–C3	9.6 ± 1.8	9.7 ± 4.3	4.5	12.88
C3–C4	9.0 ± 1.9	8.6 ± 5.9	3.2	9.75
C4–C5	9.3 ± 6.5	8.1 ± 3.8	2.8	7.4
C5–C6	6.5 ± 1.5	4.9 ± 2.1	2.6	4.07
C6–C7	5.4 ± 1.5	6.8 ± 3.3	2.2	6.91
*Axial rotation*				
C0–C1	9.9 ± 3.0	13.0 ± 5.5	5	13
C1–C2	56.7 ± 4.8	63.3 ± 13.0	56.5	60
C2–C3	3.3 ± 1.2	6.6 ± 4.8	6	4.8
C3–C4	5.1 ± 1.2	9.5 ± 4.9	4.5	5.03
C4–C5	6.8 ± 1.3	11.5 ± 3.8	4.8	6.53
C5–C6	5.0 ± 1.0	7.8 ± 4.1	4.8	3.34
C6–C7	2.9 ± 0.8	6.5 ± 3.3	5	3.06

### FE model of the ACDF procedures

**Fig. 4:**
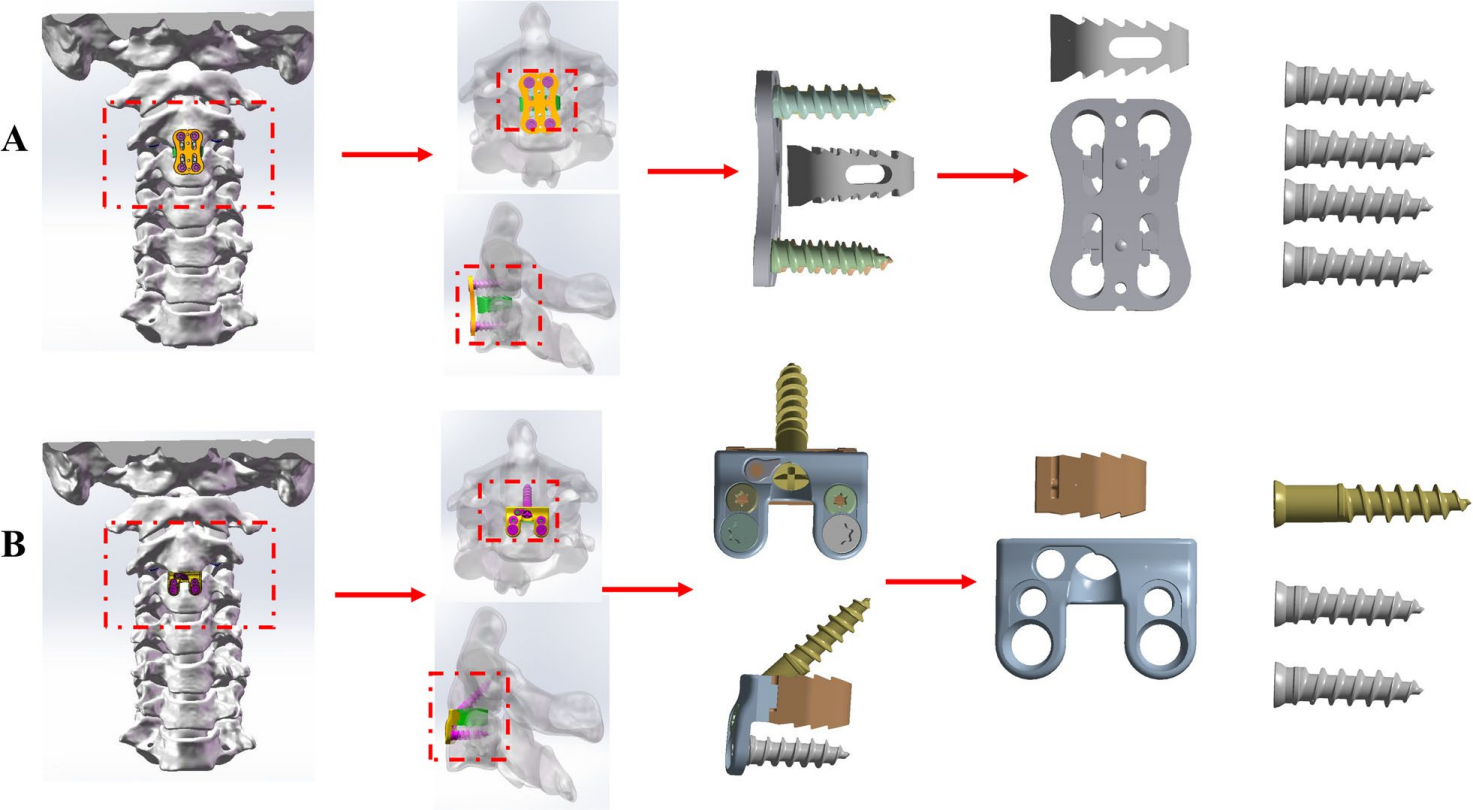
The FE models of C2–C3 ACDF using CPC (**A**) or NCCFD (**B**)

The C2–C3 ACDF model was developed based on a prior study's methodology. To summarize, the procedure involved the removal of the C2–C3 intervertebral discs along with the corresponding anterior and posterior longitudinal ligaments [13, 14] For the CPC system, PEEK cages filled with bone graft were inserted into the intervertebral space, leading to successful fusion (Fig. 4A). This was accomplished using an anterior titanium alloy plate along with four titanium alloy screws for stabilization. On the other hand, the NCCFD system utilized a plate containing bone graft that was placed within the intervertebral space (Fig. 4B). This system also achieved solid fusion through the implementation of three titanium alloy screws.

### Material property, loading, and boundary conditions

The specific material properties and element types employed in the analysis are outlined in Table 3, which are commonly utilized in the existing literature. In the static analysis, pure moments (1 Nm) were applied in the sagittal, transverse, and frontal planes, along with a compressive follower load of 50 N directed onto the superior surfaces of the occipital bone [15, 16]. Additionally, the inferior surface of the T1 vertebra was rigidly secured during the analysis.

**Table 3:** Material properties used for various components in the current FE model

Material	Young’s modulus (MPa)	Poisson’s ratio	Cross-section area (mm^2^)
Cortical bone	12,000	0.29	
Cancellous bone	450	0.29	
Posterior element	3500	0.29	
Endplates	500	0.4	
Annulus fibers	3.4	0.4	
Nucleus pulposus	1.0	0.49	
Cartilago articularis	10	0.3	
Titanium plate	110,000	0.3	
Screws	110,000	0.3	
PEEK	3600	0.3	
*Ligaments*			
Transverse ligament	20	0.25	
Cruciate ligament	20	0.3	5
Anterior longitudinal ligament (C2–C5)	30	0.3	11.1
Anterior longitudinal ligament (C5–C7)	30	0.3	12.1
Posterior longitudinal ligament (C2–C5)	20	0.3	11.3
Posterior longitudinal ligament (C5–C7)	20	0.3	14.7
Capsular ligament (C0–C1)	1	0.3	5
Capsular ligament (C1–C2)	10	0.3	5
Capsular ligament (C2–C5)	20	0.3	42.2
Capsular ligament (C5–C7)	20	0.3	49.5
Ligamentum flavum (C2–C5)	10	0.3	46.0
Ligamentum flavum (C5–C7)	10	0.3	48.9
Tectorial membrane	10	0.3	10
Interspinous ligament (C2–C5)	1.5	0.3	13.0
Interspinous ligament (C5–C7)	1.5	0.3	13.4
Supraspinous ligament	1.5	0.3	75.7
Alar ligament	5.0	0.3	20
Apical ligament	20	0.3	5
Anterior membrane	20	0.3	5
Posterior membrane	20	0.3	5

### Measurement parameters

In all three models, the ROM for each segment was assessed in different states, encompassing flexion, extension, lateral bending (both left and right), and rotation (both left and right). Moreover, in the case of both anterior fixation models, an examination was conducted on the maximum von Mises stresses experienced by the implant. This included an assessment of alterations in the Von Mises stress distributions and regions of stress concentration within the intervertebral disc and internal fixation, under varying condition states. To present the variations in ROM values and maximum stresses, histograms were created using GraphPad Prism 8.0.

## Results

### ROM of surgical models

**Fig. 5:**
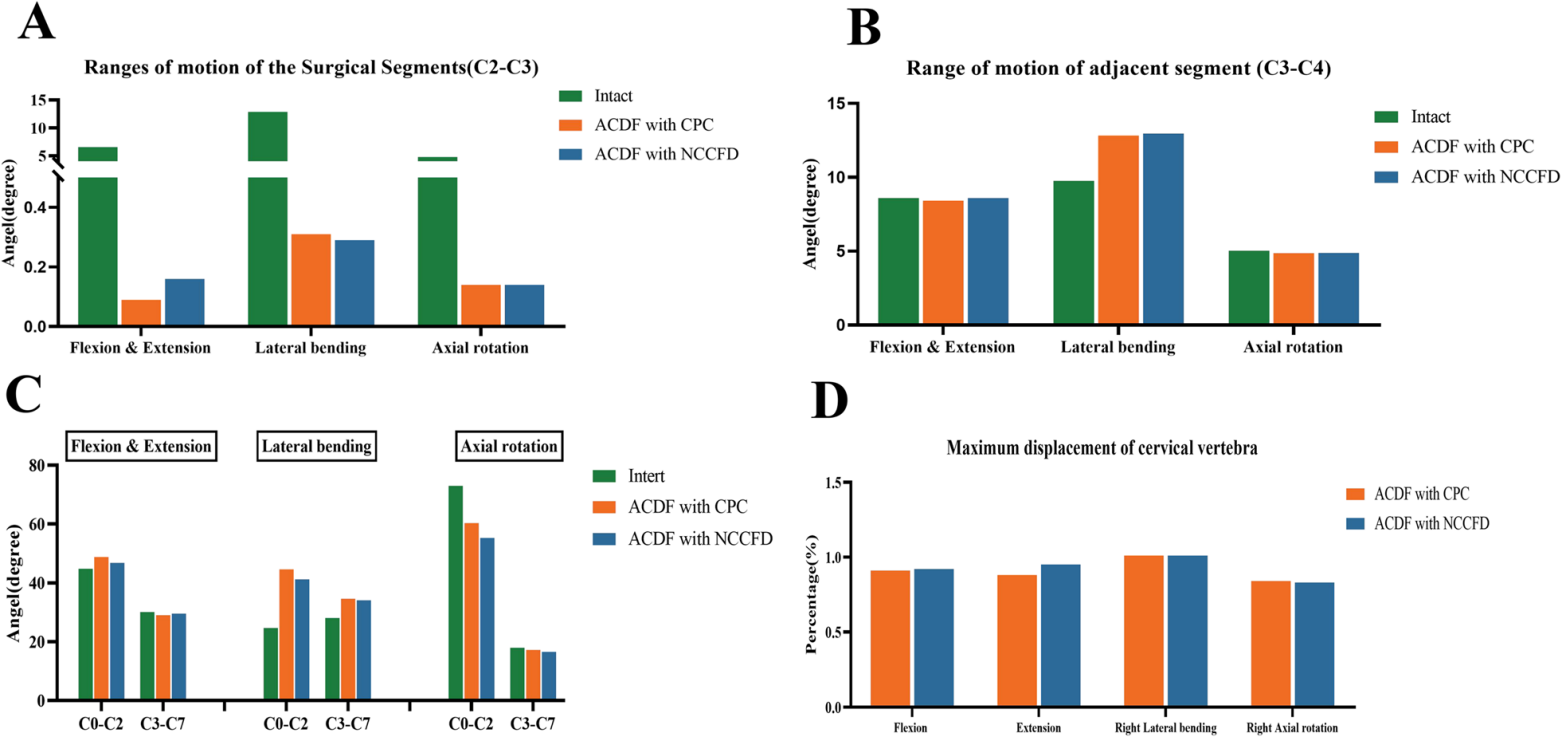
Comparison between CPC and NCCFD in ROM in C2–C3 (**A**), C3–C4 (**B**), and other segments (**C**), and maximum displacement of the cervical vertebrae as well (**D**)

C2–C3 ROM for the intact model was 6.57° in flexion and extension, 12.88° in lateral bending and 4.80° in rotation. The C2–C3 ROM decreased after ACDF in both models. Specifically, the surgical segment ROMs of CPC and NCCFD were 0.09° and 0.16°, respectively, in flexion and extension; 0.31° and 0.29°, respectively, in bending; and 0.14°and 0.14°, respectively, in rotation (Fig. 5A). The ROM of the adjacent segment (C3–C4) in the CPC and NCCFD models was increased in the lateral bending condition compared to the Intact model, which was 12.82°, 12.96°, and 9.75°, respectively. In C3–C4, the ROMs of the three models are nearly equal in flexion–extension and rotation. The ROM for adjacent segments was nearly identical for both CPC and NCCFD models when subjected to loads in all directions (Fig. 5B).

At the C0–C2 level, both the CPC and NCCFD models showed an increase in ROM in anterior flexion and posterior extension, and a substantial increase in lateral bending compared to the Intact model. However, in the rotation situation, the ROM at this region decreased. Moreover, the increase and decrease in ROM at C0–C2 in the CPC and NCCFD models were nearly identical. At both C3–C7 levels, the CPC and NCCFD models did not show a significant difference in ROM compared to the Intact model (Fig. 5C).

### Maximum displacement of the cervical vertebrae

Figure 5D illustrates the maximum displacements of the cervical vertebrae, with right lateral bending and right axial rotation serving as examples. In the CPC model, the maximum displacements in flexion, extension, right lateral bending, and right axial rotation were 0.91, 0.88, 1.01, and 0.84 times that of the Intact model, respectively. Conversely, in the NCCFD model, the maximum displacements in flexion, extension, right lateral bending, and right axial rotation were 0.92, 0.95, 1.01, and 0.83 times that of the Intact model, respectively.

### Stress at the titanium plate

**Fig. 6:**
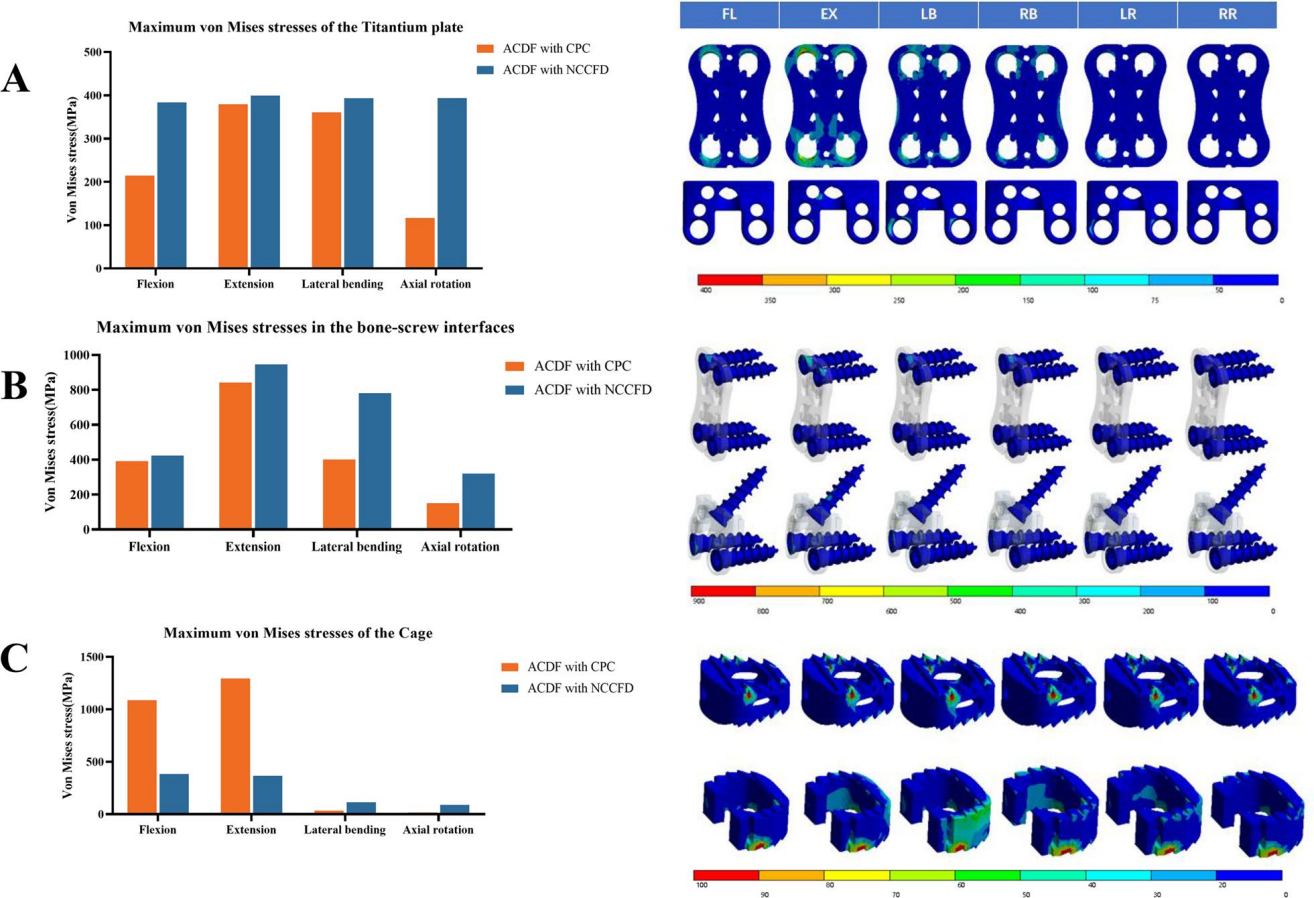
Stress at the titanium plate (**A**), bone–screw interface (**B**) and cage (**C**)

Figure 6A presents the maximum von Mises stresses experienced by the titanium plates. In the CPC model, the stresses at the anterior titanium plate were 214.6 MPa in flexion, 379.45 MPa in extension, 360.715 MPa in lateral bending, and 116.784 MPa in axial rotation. As for the NCCFD model, the stresses at the anterior titanium plate measured 383.6 MPa in flexion, 399.32 MPa in extension, 393.42 MPa in lateral bending, and 393.575 MPa in axial rotation.

### Stress at the bone–screw interface

In Fig. 6B, the maximum von Mises stresses at the C2–C3 screw interfaces are presented. In the CPC model, the stresses at the bone-screw interface were 391.68 MPa in flexion, 841.79 MPa in extension, 401.22 MPa in lateral bending, and 151.11 MPa in axial rotation. In the NCCFD model, the stresses at the bone-screw interface were 423.64 MPa in flexion, 946.20 MPa in extension, 781.14 MPa in lateral bending, and 320.15 MPa in axial rotation.

### Stress at the cage

Figure 6C displays the maximum von Mises stresses within the cages. In the CPC model, the stresses at the cage were 1087.9 MPa in flexion, 1294.6 MPa in extension, 34.679 MPa in lateral bending, and 16.4465 MPa in axial rotation. Meanwhile, in the NCCFD model, the stresses at the cage were 384.15 MPa in flexion, 366.61 MPa in extension, 114.2695 MPa in lateral bending, and 89.4695 MPa in axial rotation.

### Stress on the C3/4 intervertebral disc

**Fig. 7:**
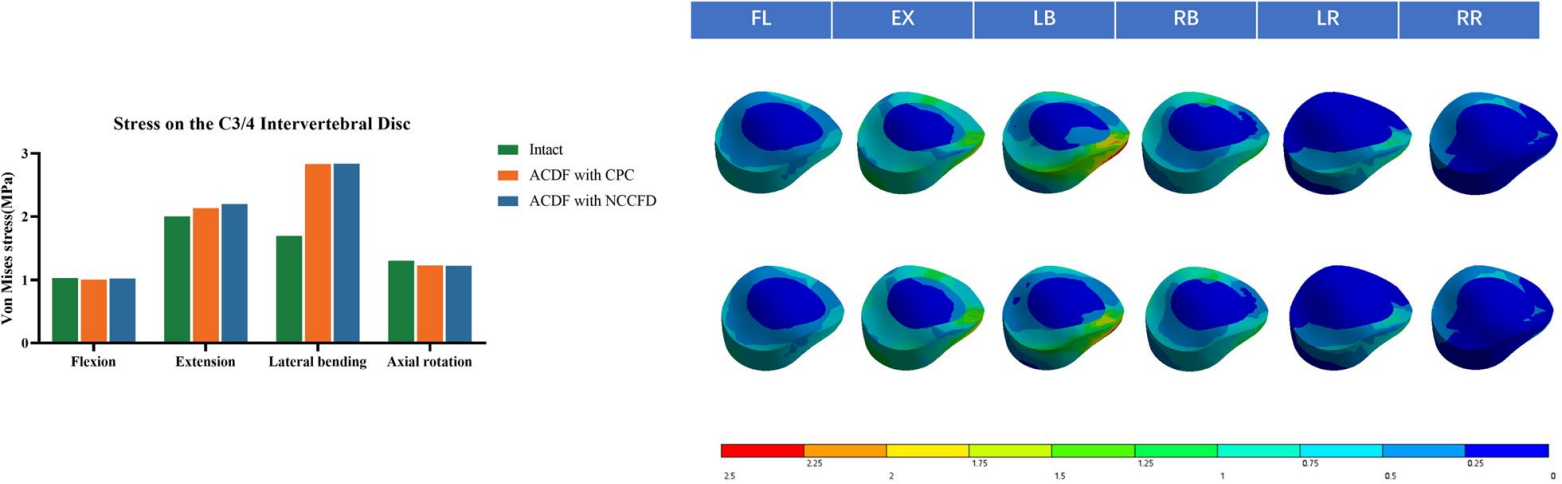
Stress on C3–C4 disc

In Fig. 7, the measurements of IDP at the supra-adjacent (C3–C4) segment are presented. In both the CPC and NCCFD models, the maximum von Mises stress values in the C3–C4 adjacent intervertebral disc were similar in flexion, extension, lateral bending, and rotation. Specifically, in the CPC model, the maximum von Mises stresses on the C3–C4 intervertebral disc were 1.0066 MPa during flexion; 2.1347 MPa during extension; 2.833 MPa during lateral bending; and 1.232 MPa during rotation. In the NCCFD model, the maximum von Mises stresses on the C3/4 intervertebral disc were 1.024 MPa during flexion; 2.1989 MPa during extension; 2.8379 MPa during lateral bending; and 1.2266 MPa during rotation. In the intact model, the maximum von Mises stresses on the C3–C4 intervertebral disc were 1.0311 MPa during flexion; 2.0035 MPa during extension; 1.6925 MPa during lateral bending; and 1.3056 MPa during rotation.

### Stress distribution at facet joint

**Fig. 8:**
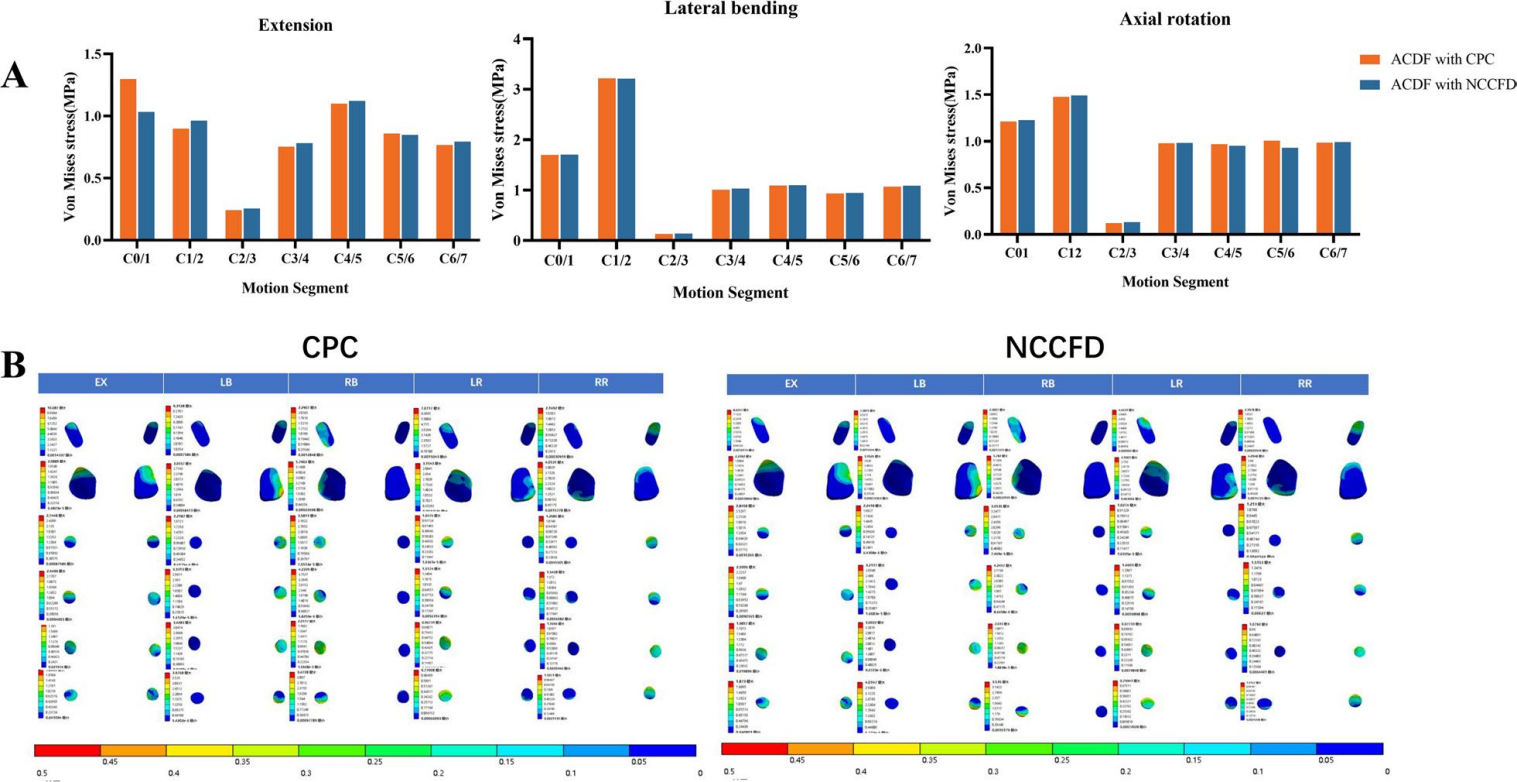
Maximum stress (**A**) and stress distribution (**B**) at facet joint under different motions

Figure 8 depicts the stress distribution at the facet joints of the C0–C7 segment. During extension, the stress was predominantly distributed anteriorly and posteriorly on both the left and right facet joints. In right bending, the stress was primarily directed anteriorly on the right facet joint, while the stress on the left facet joint was nearly negligible. Conversely, in left bending, the stress was mainly distributed anteriorly on the left facet joint, with minimal stress on the right facet joint. During right axial rotation, the stress was mainly distributed on the left side of the left facet joint, accompanied by a small amount of stress on the left side of the right facet joint. In left axial rotation, the stress was mainly distributed on the right side of the right facet joint, with a small amount of anterior stress on the left facet joint. It is noteworthy that the stress distribution in the degenerative models mirrored that of the normal models. However, while the stress distributions of the facet joints were generally similar across the three finite element models, the maximum von Mises stress values in each model varied.

## Discussion

Conventional ACDF is mainly performed with CPC and Zero-P internal fixation system to treat cervical degenerative diseases, which has good clinical value. However, the anatomical uniqueness of C2/3 is a challenge for the performance of ACDF in this segment. Therefore, an NCCFD for C2–C3 ACDF was designed in this study. In addition, the biomechanical properties of the NCCFD were evaluated in comparison with CPC systems. The focus was on factors such as ROM, IDP and stress at the endplate-cage interface. In addition, we also investigated the mechanism by which the fusion of the facet joint contributes to the acceleration of the bone fusion process.

### Construct stability

This study conducted a thorough comparison of the biomechanical stabilities offered by ACDF employing the traditional CPC and the NCCFD model. As indicated by the results, both models led to a substantial reduction in the ROM in the surgical segments when compared to the Intact model. Furthermore, the maximum displacement exhibited nearly identical values for both models. Consequently, it can be inferred that both ACDF models are capable of achieving robust construct stability in the surgical segments.

Drawing from the ROM results in the surgical segments, the NCCFD model has ROMs that are essentially comparable to the CPC model in lateral bending and axial rotation instances. In addition, under flexion and extension, the ROM of NCCFD is larger than that of CPC. We believe this might be attributed to the difference in screw number and bone-screw area, making the CPC model more rigid than the NCCFD model, resulting in lower ROM at flexion and extension. To prevent issues like intervertebral fusion device settling from affecting the results, the intervertebral fusion device's contact surface with the end plate and bone was bonded to achieve bony fusion, following Shen's study design. The establishment of bony fusion at the intervertebral space contributes to heightened stiffness within the anterior column, consequently reinforcing the stability of the construct [16]. A retrospective study conducted by Li et al. illustrated a significant reduction in ROM at the treated level following ACDF. Moreover, achieving bony fusion at the intervertebral space resulted in an additional 11.5% decrease in ROM compared to the immediate post-operative period [17]. This accounts for the higher degree of reduction in species ROM in this study compared to historical literature. The maximum displacement of the cervical vertebrae denotes the movement between two neighboring vertebral bodies, providing insight into the relative stability achieved through ACDF. The results indicate that the maximum degree of displacement for the CPC and NCCFD models is about the same as the Intact model in lateral bending, with a minor reduction in other situations. Moreover, the maximum displacements of the CPC and NCCFD models are nearly identical in each working situation. In general, the NCCFD model provides almost the same biomechanical stability as the CPC model in the treatment of C2–C3 ACDF.

### Risks of instrument-related complications

Complications associated with instrumentation, such as plate fracture, screw breakage, or cage displacement, can pose diverse risks including neck pain, compression of the esophagus, compression of the spinal cord, and in severe cases, post-operative paralysis, often requiring revision surgery. Consequently, the development of the new combined cervical fusion aimed to mitigate the likelihood of these instrument-related complications.

According to research by Lee et al. the use of a shorter plate and longer angled screws dramatically reduced ossification at the next level [18]. In this study, the volume of the traditional front neck plate was reduced by 1/2 through a design that eliminates the need to consider the curvature of the anterior edge of the C2 vertebra. This modification facilitates plate implantation, avoids blockage by the mandible during the insertion process, and mitigates swallowing difficulties caused by the plate's volume. Additionally, Kang et al. conducted a retrospective analysis of patients treated with Zero-p ACDF and concluded that deeper insertion of the titanium part at the anterior edge leads to more subsidence [19]. Consequently, this study utilized the Zero-p design, integrating the anterior cervical plate and the interbody fusion cage into a single unit, to minimize sinking of the interbody fusion cage and excessive penetration of the titanium plate into the intervertebral disc.

The anterior cervical plate and screw of the CPC achieve a better-balanced maximum stress in all circumstances, as observed in the implant maximum stress. This design addresses the typical anterior cervical plates' issues, which experience high lateral bending stress in extension and low rotational stress in anterior flexion. Although the maximum stress of NCCFD screws in lateral bending and rotational conditions is significantly higher than that of the CPC, the NCCFD model's maximum stress remains within an acceptable range [20]. This is because the NCCFD model uses one less screw than the CPC, and the maximum stress point is concentrated at the junction of the upper screw and the anterior plate, staying within the tolerance range of the titanium alloy. Comparing the maximum fusion stress, it was found that the CPC experiences much lower fusion stress in anterior flexion and extension than the NCCFD, with no significant difference between the two in lateral bending and rotational states. This is due to the design integration of the anterior cervical plate with the intervertebral fusion, which improves force transmission to the vertebral body below, preventing issues like settling and dislocation of the intervertebral fusion due to excessive force and adapting to the cervical spine's force transmission.

### Risks of the degeneration at adjacent intervertebral disc and facet joints

Assessing changes in internal stresses at adjacent levels of surgical segments through measuring the IDP is crucial [21]. Elevated IDP at adjacent levels can contribute to ASD, potentially affecting a patient's postoperative recuperation and overall well-being. These heightened IDP levels following surgery may arise from a range of factors, including disc-related issues, alterations in cervical curvature, and ensuing discomfort [22].

Comparing the maximum stresses in the CPC and NCCFD discs, it was observed that maximum stresses rise in extension and lateral bending for both models. This is attributed to the loss of mobility in the fixed segment during top-to-bottom force transmission, leading to increased stress on the adjacent segment. Furthermore, when a lateral bending motion occurs, the stabilized segment experiences limited movement, causing the neighboring segment to become more mobile. As a consequence, there is heightened pressure exerted on the intervertebral disc within that neighboring segment. The stress on the intervertebral disc at C3–C4 remained largely consistent in both models across various operational scenarios. This suggests that our recently developed intervertebral fusion had a comparable impact on adjacent segments when compared to the cage-plate construct.

In the current study, joint surface stresses for both types of internal fixation were much lower in the surgically fixed segments, consistent with Shen's findings [16]. The stress at the joint surface of the upper cervical spine increased in all situations, while the stress at the joint surface of the lower cervical spine remained practically the same as that of the normal cervical spine in all situations. This phenomenon arises from the support of the head by a two-column structure at the C1 vertebral level, which transitions into a three-column structure at C3. As a result, load transmission primarily occurs through the vertebral bodies rather than the facets. This redistribution of loads takes place within the C2 vertebra [23]. However, in both the CPC and NCCFD models, there is firm fixation of the anterior column of the C2–C3 cervical spine with limited movement of the two posterior columns. As a result, forces that would have been carried from the C2 to the C3 posterior column could not be smoothly transmitted, resulting in higher stresses on the upper cervical spine's joint surfaces.

Moreover, the robustness of the paravertebral muscles holds a pivotal role in modulating IDP and stress on the Facet Joints. Hence, patients should be mindful of their neck muscle strength and consider incorporating exercises into their daily routines. This practice can help mitigate elevated IDP at adjacent levels following surgery.

### Clinical relevance

Performing ACDF using the CPC system at C2–C3 levels presents specific challenges and complexities due to the unique anatomical characteristics of this region. Firstly, it is a complex surgical approach. The anatomy of the upper cervical spine makes access and visualisation more difficult than at lower levels. The proximity of the mandible, hyoid and tongue can make it difficult for the surgeon to access the surgical site. In addition, the limited exposure and working space at the C2–C3 level makes disc removal, implant placement and proper fixation more difficult than at lower levels. In addition, the C2 vertebra has a unique anatomical structure with the odontoid process, which can complicate the surgical approach and implant placement. Additionally, it has been noted that the raised edge of the plate has a tendency to exacerbate irritation to the soft tissues of the pharynx and esophagus, resulting in postoperative dysphagia. The larger size of the plate also requires more removal of prevertebral soft tissues, increasing the likelihood of injury to the superior laryngeal nerve. This potential nerve damage further complicates the surgical process and may result in adverse postoperative effects.

The NCCFD system provides a solution that effectively addresses the limitations of the CPC system, particularly when used at the C2–C3 level. Importantly, this system achieves a significant reduction in the size of the conventional anterior cervical plate by half. This adjustment is critical to accommodate the limited exposure and working space inherent at the C2–C3 level. In addition, the system features three screw holes, with the top screw angled at 35°–70° to accommodate the unique anatomical configuration of the C2 vertebra. This intricately designed system is specifically tailored to address the unique anatomical challenges of ACDF procedures at the C2–C3 level. Its objectives include providing enhanced stability, reliable fixation and appropriate interbody support. This innovative approach is designed to improve surgical outcomes and potentially reduce the complications commonly associated with conventional ACDF techniques. Based on the results of the finite element analysis performed in this study, the NCCDF demonstrated comparable biomechanical stability to the CPC. It is important to emphasise that although biomechanical data should not be extrapolated beyond the immediate postoperative period, the results presented in this study support the continued clinical use of the NCCDF in cervical spine fusions at the C2–C3 level. The unique design of the NCCDF effectively mitigated the challenges associated with anterior upper cervical surgery, allowing young orthopaedic and neurosurgeons to explore more accessible and efficient solutions for upper cervical spine disorders. It also addressed the issue of limited specialized anterior plates for C2/3 and was also suitable for anterior surgery in cases of short neck deformity, mandibular hypertrophy and similar conditions. Ultimately, the NCCDF represented a new option for anterior surgical instrumentation in the upper cervical spine.

### Limitation

Indeed, this study has some limitations that should be considered. Firstly, the material attributes were simplified, with ligaments represented by linear springs and other structures treated as solid constructions. Additionally, the structural simplification of the cervical spine in the study. Muscles were not accurately replicated, and the study could not fully simulate the cervical spine’s activity in all directions under real-life conditions. Furthermore, this incompleteness of the ASD risk assessment should be addressed as the FE model was constructed without considering gender differences or degenerative changes such as articular synostosis, endplate sclerosis, annular disc tears or vertebral body osteoporosis.

## Conclusions

In conclusion, this finite element study comprehensively analyzed the biomechanical changes in instrumentation, adjacent intervertebral discs, facet joints, and internal fixation during the fusion process using both CPC and NCCFD internal fixation methods. NCCFD not only provides the same level of biomechanical stability as CPC but also avoids postoperative complications associated with uneven force damage to the implant. The device offers a novel surgical alternative for ACDF in C2–C3 level, which can be attempted at other segments in the future.

## Data Availability

The datasets generated during and analyzed during the current study are not publicly available due to them containing information that could compromise research participant privacy/consent, but are available from the corresponding author on reasonable request.

## Abbreviations

FE: Finite element
ROM: Range of motion
IDP: Intervertebral disc pressure
